# Size Effect on Mechanical Properties and Texture of Pure Copper Foil by Cold Rolling

**DOI:** 10.3390/ma10050538

**Published:** 2017-05-16

**Authors:** Meng Song, Xianghua Liu, Lizhong Liu

**Affiliations:** 1 The State Key Laboratory of Rolling and Automation, Northeastern University, Shenyang 110819, China; som1128@foxmail.com; 2 Research Academy, Northeastern University, Shenyang 110819, China; 3 School of Materials and Metallurgy, Northeastern University, Shenyang 110819, China; liulizhong@smm.neu.edu.cn

**Keywords:** size effect, ultimate strength, pure copper, cold rolling, surface grains

## Abstract

To study the size effect on the properties of copper, tensile tests were performed with pure copper foil (thickness range from 25 μm to 300 μm) by cold rolling. A pronounced size effect was observed at a thickness of 76 μm. The results showed that ultimate strength increased as sample thickness decreased from 300 μm to 100 μm, however, this was decreased as the thickness changed from 76 μm to 25 μm with ultrahigh strain, with the same trend of dislocation density and micro stain. The rolling texture were consisted of copper {112}<111>, brass {011}<211>, and S {123}<634>. These features seemed to be linked to the increase of fraction of surface grain to volume, which led to lower districting on the dislocation slip.

## 1. Introduction

Presently, many researchers are interested in the mechanical properties of materials in micro-scale, due to a significant increase in demand for miniaturization production and multi-functional integration. To fabricate micro-scaled parts efficiently and accurately, the mechanical behavior in micro-scaled deformation is a significant issue to be explored and in-depth understanding of those behaviors urgently needs to be established [1]. 

In micro-scale deformation, mechanical responses have been observed to be size effect, which are different from behaviors in a macro-scale. Numerous reports have concluded that the size effects follow the trend of smaller meaning stronger. Polycrystalline thin films on substrates was investigated extensively decades ago [2], and the increasing in strain hardening of thinner films was closely related to the confinement of dislocation generation and movement within a small volume of grain. It was reported for uniaxial compression on gold pillars of diameters varying between 300 nm and 7450 nm, that the pillars yielded at stresses that were much higher than the typical yield strength of bulk gold of ~30 MPa at 2% strain. This appeared to be controlled by dislocation starvation [3]. Similar results were recently reported for single crystal pillar compressive experiments [4,5,6].

However, the size effect appears as the smaller the weaker in the thickness range from a few to hundreds of microns. H. Hoffmann et al. [7] investigated the flow stress with different thicknesses from 500 μm to 10 μm with different grain sizes, and showed the flow stress curve scaled down below a thickness of 200 μm. Creep deformation on the freestanding aluminum nano-film showed that the steady-state creep rate increase as the thickness from 800 nm to 400 nm, and then decreased in the 200–100 nm thickness range [8]. As the film thickness decreased to 200–100 nm, the driving force to reduce the surface area due to surface tension became dominant, leading to a decrease in the creep rate. Compression deformation behavior of nanocrystalline copper pillars with an average grain size of 360 nm to 34 nm has been investigated as a function of specimen size [9], where the yield stress of 360 nm and 100 nm exhibited essentially bulk yield stress until the specimen sized down to critical values (specimen/grain size = 35 and 85), below which the yield stress decreased with a decrease in specimen size. Dislocation glide within crystalline grains was the dominant deformation mechanism for Cu pillars with grain sizes of 360 nm to 10 nm, while grain boundary creep was dominant for pillars with a grain size of 34 nm. 

Furthermore, size effect is analyzed by the ratio of sample thickness to grain size T/D. The fatigue life of the Cu foils strongly depended on the T/D, and surface grains gradually became a dominant factor to control fatigue properties [10]. Chen et al. [11] carried out tensile tests with the Ag wires of diameters from 20–50 μm and grain size 3.5–40.6 μm, and found that the strengthening effect depended on shape as the T/D decreased from ~3. This meant that the size effect existed with a smaller T/D. Uniaxial tensile was performed on the brass sheet with thickness in the range of 50–200 μm with different T/D, and the flow stress increased with increasing T/D for larger values of T/D and decreased when the values of T/D were less than 3 [12]. Similarly, Wang et al. [13] found that the Hall–Petch was not valid and that even an inverse Hall–Petch effect occurred as the T/D decreased from 6.2 to 3.5. This effect was attributed to the competition of free surface weakening and external boundary strengthening. 

Several theoretical models have been proposed in order to explain the size effect, e.g., strain gradient theories [14], discrete dislocation dynamics theories [15], and fracture mechanism theories [16], whose models were based on the microstructures, which were investigated by discrete dislocation dynamics simulations in recent years [17,18]. 

In summary, different kinds of size effects have been reported in the literature. The elastic, plastic, and failure properties seemed to be dependent on both the sample size and grain size. Despite numerous investigations, some fundamental questions concerning the size dependency of mechanical properties remains open. First, only a few investigations have dealt with the size effect on the rolling of foil, especially in the negative roll gap; and second, almost nothing is known about the geometry size effect on the mechanical properties with ultrahigh strain. 

Hence, the aim of the present work was to perform a detailed investigation on mechanical properties using a uniaxial tensile test with different thicknesses of commercial pure copper (pure copper) ranging from 300 μm to 25 μm due to asymmetrical cold rolling (ASR). It is helpful to understand the mechanism of size effect on the foil rolling to optimize the micro manufacturing process and improve the quality of micro parts.

## 2. Materials and Methods

The study was carried out on pure copper in plate form with dimensions of 5.8 mm × 35 mm × 35 mm (thickness × width × length), obtained after isothermal annealing at 600 °C/60 min. The chemical composition is listed in Table 1. 

The material, in the initial state, was subjected to main cold rolling to a final thickness 25 μm obtained in two stages without intermediate annealing at room temperature. First, it was rolled to 1000 μm on a laboratory two high mill with a work roll diameter of 180 mm, and second, it was rolled to a final thickness 25 μm by micro metal forming mill (3M mill).

**Figure 1:**
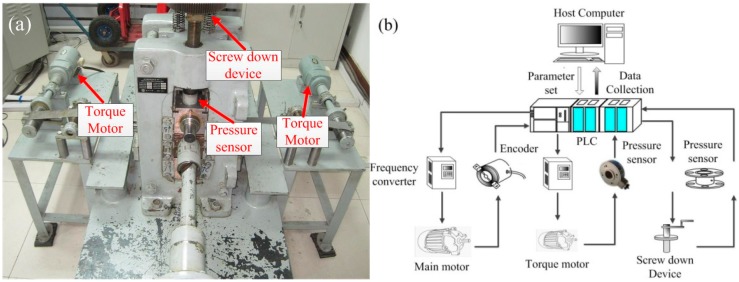
Illustration of (**a**) 3M asymmetric mill and (**b**) the control system.

The 3M mill is a new type of rolling mill with an ultra-thin rolling capacity developed by our research group and is shown in Figure 1. The main parameters and characters are as follows: backup roll diameter: 120 mm, work roll diameter: 50 mm, roll barrel length: 130 mm, maximum roll force: 200 kN. The speed ratio between the up and down rolls can be changed from 1 to 1.3 continuously.

Uniaxial tensile tests were performed on an Instron 5969 microforce Testing System (Illinois Tool Works Inc., Cincinnati, OH, USA) at a constant strain rate 10^−4^ s^−1^ at room temperature. The extremely high precision ensured the accuracy of the test data, especially for the ultrathin samples. The major device parameters were as follows:

The resolution of testing force: 1/500,000 FS (Full scale).
Maximum data acquisition frequency: 2500 Hz.
Repetitive precision of beam position testing: <2 μm.
Automated extensometer: (automate griped and automate opened) resolution: 0.1 μm, absolute accuracy: ±1 μm.

**Figure 2:**
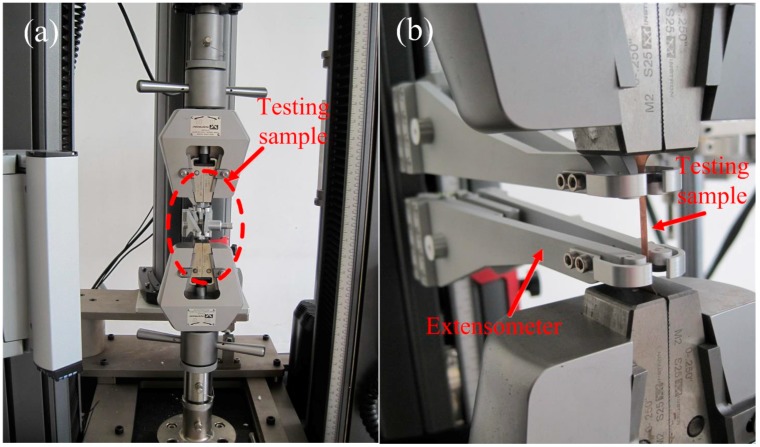
Details of (**a**) uniaxial tensile test and (**b**) the extensometer on microforce testing system.

Dog-bone-shaped tensile specimens with a gauge length of 50 mm and width of 3 mm were prepared by electro discharging from the rolled Cu foils (see Figure 2). The final sample thickness for tensile strength after ultrasonic cleaning for ~10 min in 80% acetone, measured at least 10 points by a CHY-CA thickness tester (Labthink Instruments Co., Ltd., Jinan, China) with a resolution ratio of 0.1 μm. To prevent slipping of the Cu foil in the tension test, sandpaper should be added at the clamped ends. The tensile test was carried out at least five times for each Cu sample.

The microstructure of the deformed samples was investigated by transmission electron microscopy (TEM). The TEM study was carried out using a Tecnai G220 transmission electron microscope (FEI Technologies Inc., Hillsboro, OR, USA) operated at 200 kV. The observation sections were perpendicular to the transverse direction (TD) and normal direction (ND) of the rolled foil. To perform a systematic TEM investigation, a special technique for the preparation of thin foils was implemented, which was the focused ion beam (FIB, FEI Technologies Inc., Hillsboro, OR, USA) technique. 

The macroscopic texture was measured in a Schulz back reflection method on a semiautomatic texture measuring table by X’ Pert Pro X ray diffraction (PANalytical B.V., Almelo, The Netherlands) at 35 kV, and represented by orientations distribution functions (ODFs), which were calculated by the two-step method, and the results were shown by constant ψ sectional view (Δψ = 5°), the maximums of ψ, θ, φ were 90°.

**Figure 3:**
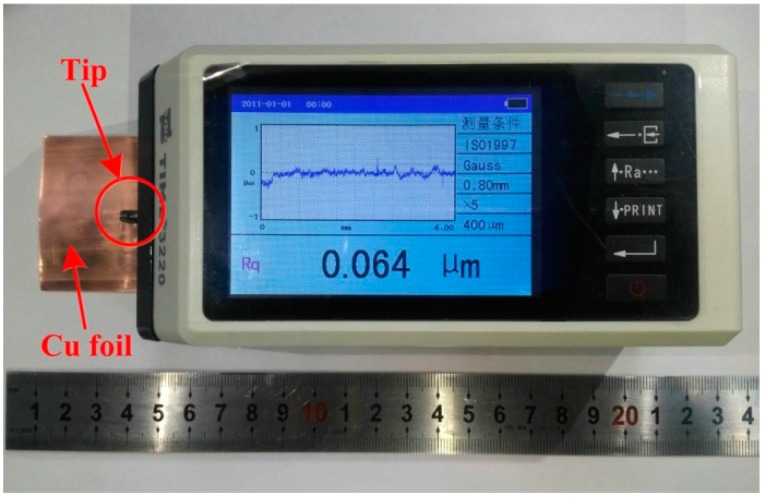
Testing of surface roughness for Cu samples of 76 μm.

The surface roughness of the Cu samples was measured by a high precise roughmeter, which is shown in Figure 3. The radius of the test tip marked by the red row in Figure 3 was 2 μm, testing force of the stylus was 0.75 mN, and the resolution was 0.001 μm. The measured surface roughness was the root mean square value of the ordinate in 0.800 mm sampling length, and the measured value was expressed as

(1) $$Rq=\sqrt{\frac{1}{l}\int_{0}^{l}Z^{2}\left(x\right)dx}$$

where the Rq was the root mean square value of surface roughness, Z(x) represented the ordinate in sampling length, and l was the sampling length.

## 3. Results

### 3.1. Tensile Behavior

**Figure 4:**
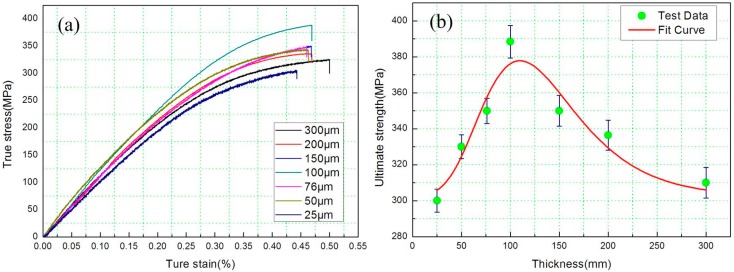
(**a**) Tensile true stress–strain curves; and (**b**) ultimate strength vs. thickness for commercial pure copper of thicknesses from 25 μm to 300 μm.

True stress–strain curves of samples with thicknesses from 25 μm to 300 μm are depicted in Figure 4a. When the Cu sample was deformed by micro rolling, dislocations were generated, moved, and stored; the storage caused the materials to work harden. Therefore, the ultimate strength was enhanced from 307 MPa to 388 MPa, as the thickness of the Cu samples was decreased from 300 μm to 100 μm and the stress strain curves almost covered each other. However, a pronounced size effect was found when it was thinner than 76 μm: the ultimate strength decreased from 350 MPa to 300 MPa as thickness decreased from 76 μm to 25 μm with further deformation, that is, the thinner foils had a lower ultimate tensile strength. Elastic deformation was affected by the size effect, that is, a Young’s modulus defect in deformed metal was found when thicknesses of Cu samples were down from 100 μm to 25 μm. The Young’s modulus defect has been reported by several authors [19,20,21,22]. The most relevant contribution to the modulus defect seems to be the reversible motion of dislocations. Hordon et al. [23] claimed that the modulus defect should approximately be proportional to $N\cdot d^{3}$, where N is the dislocation density and d is the average distance of a dislocation segment between two pinned points. Since the grains of the Cu foils were not embedded in a three-dimensional matrix, the constraints along the grain boundaries on the surface grains are relaxed [24]. The surface grains significantly increased from 76 μm to 25 μm, therefore, the modulus defect was found. A remarkable work softening from 76 μm to 25 μm is displayed clearly in Figure 4b, and are rarely observed in other heavily cold worked materials [25,26,27]. Furthermore, the fit curve in Figure 4b was obtained by the function of Nonlinear Curve Fit of Origin software. The fit equation was

(2) $$y=y_{0}+Ae^{\left(-e^{\left(-z\right)}-\frac{x-xc}{w}+1\right)}$$

where the $y_{0}$, xc, w, A were 302.04, 109.76, 48.34 and 75.81, respectively.

**Figure 5:**
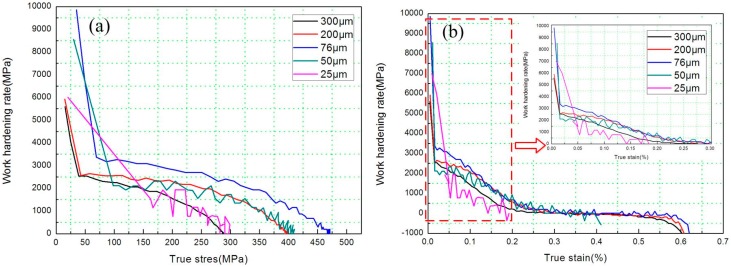
(**a**) Work hardening rate plotted vs. true stress and (**b**) true strain for commercial pure copper of thicknesses from 25 μm to 300 μm.

The work hardening rate of pure copper samples was defined in [28,29] is

(3) $$\Theta =\frac{d\sigma }{d\varepsilon }$$

where σ and ε are the macroscopic true stress and true plastic strain, respectively. Θ is a derivative of a state function σ(ε). Based on the afore-mentioned true stress–strain curves, Θ was plotted vs. the true stress and strain for the commercial pure copper of thicknesses from 25 μm to 300 μm (Figure 5a,b, respectively). All curves in Figure 5 displayed an initially steep decrease in Θ for strains of less than ~0.2%, which corresponds to the elastic-plastic transition. Subsequently, there was an approximately linear decrease of Θ as the true strain increased, analogous to the conventional stage Ⅲ hardening described by the Kocks–Mecking model [30,31]. The sample with a thickness of 25 μm offered a distinctly reduced hardening rate at both higher stress and higher strain. Comparing the hardening rate values of the other samples, which were thicker than 76 μm, it was clear that the heavier strain enhanced Θ appreciably. However, the phenomenon was different from the above-mentioned when the sample thickness was below 76 μm, which is consistent with the tension results in Figure 4.

### 3.2. Microstructure Evolution

**Figure 6:**
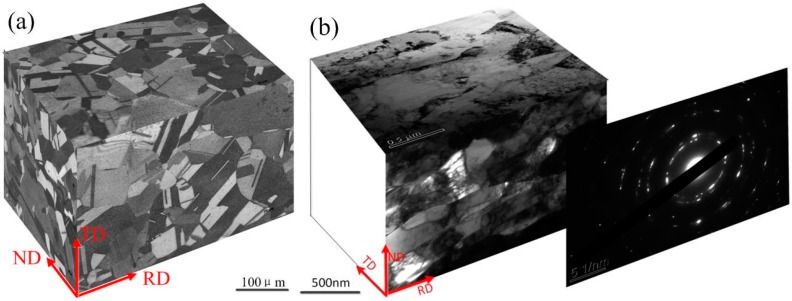
Optical Microscope (OM) and Transmission Electron Microscope (TEM) micrographs in specimen of (**a**) as-received with thickness of 5800 μm; and (**b**) processed by cold rolling with a thickness of 25 μm.

**Figure 7:**
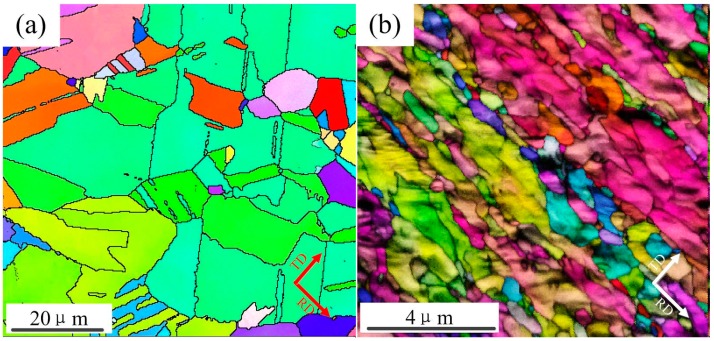
Electron Back Scattered Diffraction (EBSD) mapping representing the microstructure in the sample of (**a**) as-received with thickness of 5800 μm; and (**b**) processed by cold rolling with a thickness of 25 μm.

Figure 6a,b shown typical optical microscope (OM) and transmission electron microscope (TEM) morphologies of pure copper, which was as-received with a thickness of 5800 μm and processed by cold rolling with a thickness of 25 μm, respectively. In addition, Figure 7a,b display electron back-scattered diffraction (EBSD) mapping of the above sample. 

The pure copper as-received consisted of roughly equiaxed grains with an average size of ~60 μm, and contained a typical annealing twin band ~20 μm (see Figure 6a) with random orientations (see Figure 7a). The microstructure obtained after ultrahigh strain subject to cold rolling to 25 μm without intermediate annealing at room temperature (see Figure 6b), was revealed by TEM investigation to have a significantly finer substructure which contained fine twin lamellae, thin laths, and elongated subgrain structures. These ultrafine (sub)grains possessed a range of dimensions varying from ~80 nm to 200 nm, and were almost completely parallel to the rolling direction (RD). Some of these (sub)grains appeared to contain few dislocations and were delineated by relatively sharp boundaries. The selected area diffraction (SAD) patterns obtained from the above micro-region displays a circle which indicates high angle misorientations of neighboring areas of the crystal lattice, which was consistent with the EBSD mapping (see Figure 7b).

### 3.3. Calculation of Dislocation Density

**Figure 8:**
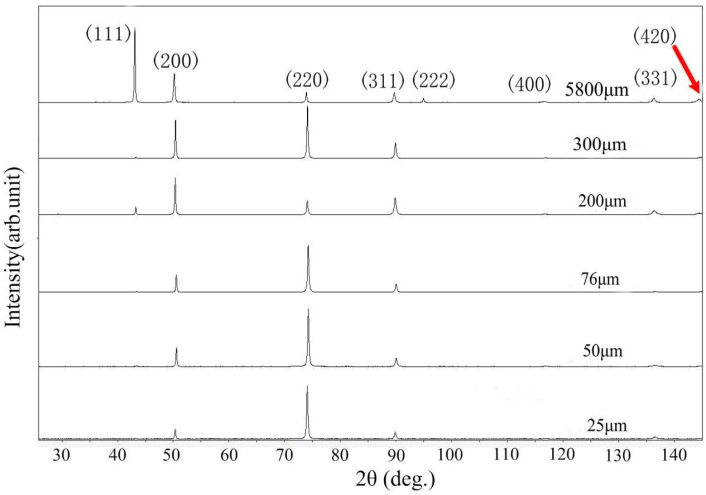
Comparison between the XRD of the annealed pure copper and after rolled.

The XRD patterns of an annealed pure copper and the after cold rolling with different extensibility in thickness ratio (ETR), can be written as η [32] (Figure 8).

(4) $$\eta =\frac{H}{h}\times 100\%=\frac{b}{B}\left(\frac{l-L}{L}+1\right)\times 100\%\approx \left(\delta +1\right)\times 100\%$$

where B and L are the sample width and length before forming; b and l are the width and length after forming, respectively. As the spread during the cold rolling was very little in many cases, it could take b≈B. A significant decrease in peak intensity and their broadening due to very small grain size and a high level of internal elastic strains are the features of the XRD pattern for the rolled Cu sample. The increased of deformation led to a gradual decrease in intensity of the peaks for (111) planes, (200) planes, (311) planes, (222) planes, (400) planes, (331) planes, and (420) planes. Moreover, the additional asymmetrical broadening and increase of the (220) planes diffraction peak on the XRD pattern may have appeared due to a high density of grain boundary dislocation [33], which corresponds to the TEM observation in Figure 6b. The internal breadth analysis was used to calculate the grain size and microstrain from the XRD line broadening. The mean grain size for the 25 μm samples was approximately 150 nm, which was consistent with the EBSD mapping in Figure 7b. This analysis presumes that grain size broadening and strain broadening profiles could be approximated by Cauthy and Gaussion functions, respectively, leading to the equation [34,35]

(5) $$\frac{{\left(\delta 2\theta \right)}^{2}}{\tan^{2}\theta_{0}}=\frac{\lambda }{d}\left(\frac{\delta 2\theta }{\tan\theta_{0}\sin\theta_{0}}\right)+25\langle e^{2}\rangle$$

where δ2θ is the measured integral breadth, $\theta_{0}$ is the peak maximum position, λ is the wave length, d is the average crystallite size, and e is the microstrain. 

The dislocation density $\rho_{s}$ calculated from the strain broadening was [36]

(6) $$\rho_{s}=\frac{k\varepsilon^{2}}{Fb^{2}}$$

where k is a constant of 4, b was the Burgers vector, and F is a strain energy factor. The dislocation density $\rho_{p}$ calculated from the grain size was [36]

(7) $$\rho_{p}=\frac{3n}{d^{3}}$$

where d is the grain size, and the n was the number of dislocations per grain block face. The inequality of Equations (6) and (7) indicated the probable dislocation arrangement and in the case of a dislocation piled up array F equals n, the number of dislocations in the pile-up, and 

(8) $$\rho_{ture}={\left(\rho_{s}\rho_{p}\right)}^{\frac{1}{2}}={\left(\frac{12\varepsilon^{2}}{d^{2}b^{2}}\right)}^{\frac{1}{2}}$$

**Figure 9:**
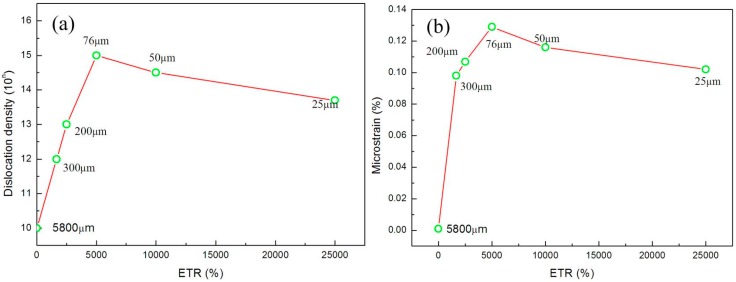
(**a**) Dislocation density and (**b**) microstrain plotted vs. different extensibility in thickness ratio (ETR) for the cold rolled pure copper.

For the materials processed by cold rolling, the dislocation density ρ could be represented in terms of grain size d and micro strain ${\langle \varepsilon^{2}\rangle }^{\frac{1}{2}}$ by the equation [36,37,38]

(9) $$\rho =\frac{2\sqrt{3}{\langle \varepsilon^{2}\rangle }^{\frac{1}{2}}}{d\times b}$$

where b is the Burgers vector and equals to 0.256 nm for a pure Cu. The dislocation density and microstrain of the initial Cu and after cold rolled Cu with different extensibility in thickness ratio (ETR) are shown in Figure 9. It can be seen that the dislocation density increased initially when thickness was greater than 76 μm and then decreased when thicknesses were less than 76 μm, as well as the microstrain variation tendency. 

### 3.4. Texture Evolution

**Figure 10:**
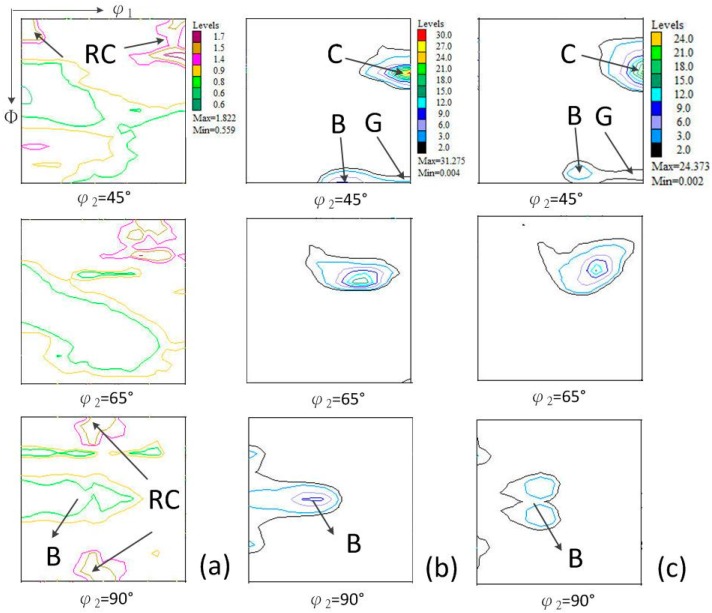

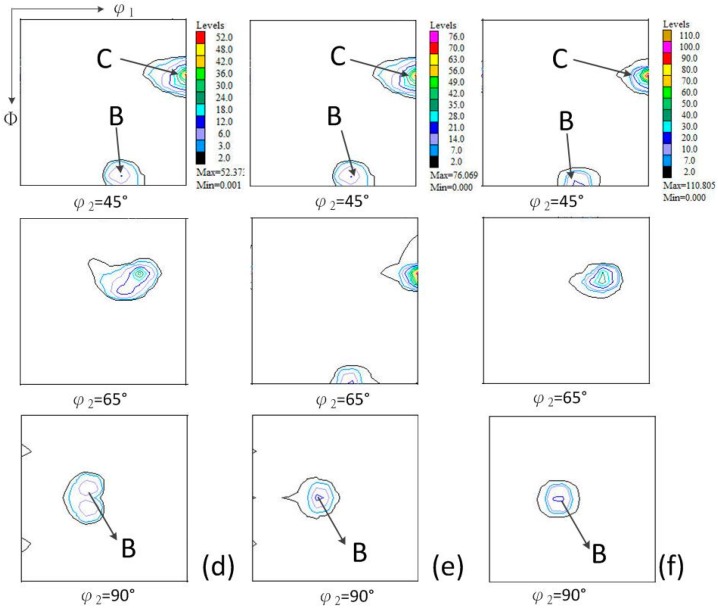
$\varphi_{2}$ = 45°, 65°, and 90° ODF sections obtained for pure copper in (**a**) initial state; and after asymmetrical cold rolling to (**b**) 300 μm; (**c**) 200 μm; (**d**) 76 μm; (**e**) 50 μm; (**f**) 25 μm texture components in considered Euler angle space section.

**Figure 11:**
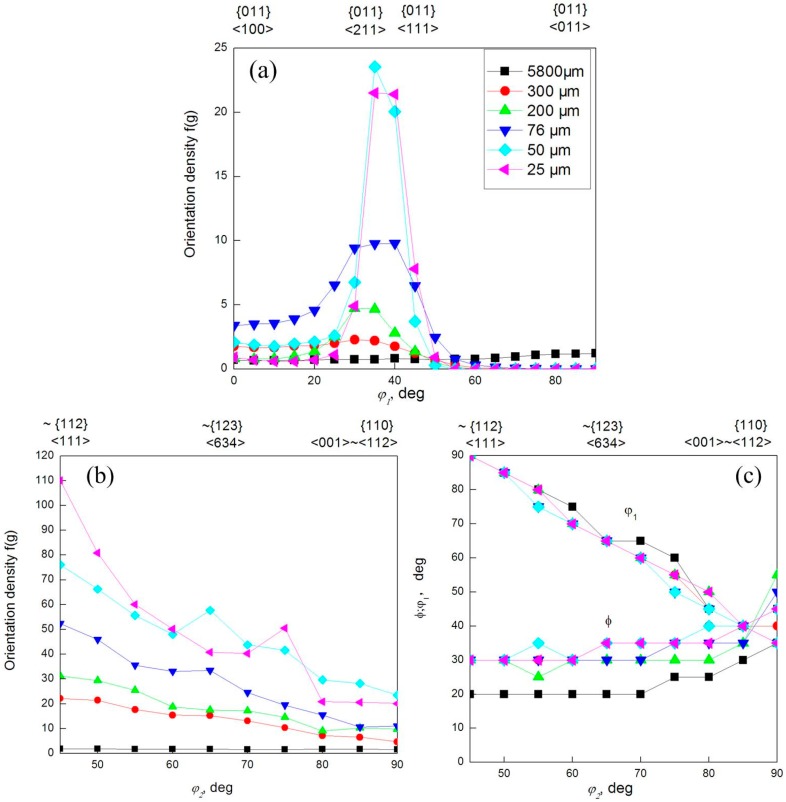
(**a**) α-fiber; (**b**) β-fiber; and (**c**) β-fiber position plots for pure copper in initial state and different thicknesses after asymmetrical cold rolling.

Figure 10 shows the orientation distribution function graphs of surface of Euler angles space for the $\varphi_{2}$ = 45°, 65°, and 90°, calculated for pure copper with different thicknesses from 5800 μm to 25 μm. In addition, Figure 10 shows the α-fiber, β-fiber, and β-fiber position courses in the Euler angle space, respectively. Material before rolling treatment (see Figure 10a) had a texture dominated by the rotate cube component {100}<011>, which is a basic component of recrystallization textures in pure FCC metal with medium to high stacking fault energy [39]. Ultra high strain cold rolling from 5800 μm to 25 μm led to a significant change and sharpening of texture [40]. For asymmetrical rolling copper (thickness 300 μm, Figure 10b), the maximum value of the ODF was nearly 20 times greater than for the starting material, whereas in the case of further asymmetrical rolling samples (see Figure 10c–f) was about 50 times greater. Pure copper rolled with equal speed of both rolls and asymmetrical ratio 1.2, had a typical rolling texture of pure metal type, which is formed by the copper (C) {112}<111>, brass (Bs) {011}<211>, and the S {123}<634> (observed in Figure 11). Increasing of the strain led to further rolling texture sharpening, with a noticeable rise in intensity of the rolling texture component {011}<211>, instead of typical shear texture {001}<110>, {111}<110>, and {111}<112>, that may be the texture genetic effect of symmetrically rolling from 5800 μm to 76 μm. 

## 4. Discussion

In this study, there was a clear dependence of the mechanical behavior on the thickness of the pure copper foils in the tensile test: thinner foils displayed a smaller ultimate strength than thicker ones processed by asymmetrically + micro rolling. According to Xu [16], the size effect of materials can be understood from the grain size and geometry. To explain the above-mentioned findings, the combination of several mechanisms and effects should has to be considered, as well as the differences in geometry and microstructure between samples of different thickness. 

**Figure 12:**
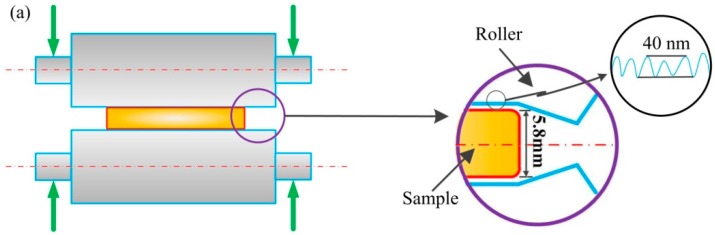

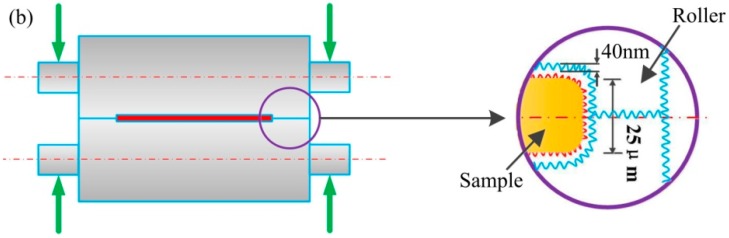
Difference of (**a**) normal gap rolling; and (**b**) negative gap rolling.

Due to micro rolling, the geometry scale of pure copper spanned from macro to micro, where it was rolled from 5800 μm to 25 μm without intermediate annealing at room temperature. The notable character of micro rolling was the negative roll gap, that is, the elastic contact deformation between the work roll, which remained outside the loaded roll gap. Figure 12 shows the difference between normal gap rolling and negative gap rolling. It was clear that the rolls bent elastically without contacting each other in the normal rolling gap (Figure 12a), in contrast, the roll elastic contacted each other apart from flattening in negative rolling gap (Figure 12b). Furthermore, the transverse compression, which significantly improves the plasticity of pure copper [41], was increased by the negative gap rolling [42], and may be due to more active slip systems. In the different rolling process, the friction was increased as the thickness varied from macro to micro, which is in agreement with the literature [43]. The surface roughness of the samples ranged from 300 μm to 25 μm were listed in Table 2. It can be clearly seen that the surface roughness is increased as the thickness decease, which is in agreement with the literature [44,45].

When the work roll pressed the lubricated material surface, the roughness peaks deformed plastically. Lubricant could be squeezed out, and the so-called open lubricant pocket (OLP) was thus formed [43]. The increase of surface roughness was due to the increase of the area fraction of OLP, which accelerated the wear of the work roll in the micro rolling process. In addition, the change of friction coefficient may be attributed to the fact that the surface asperities on the tooling and sample were not scaled down with the sample size, and the ration asperities to the sample size increased with micro rolling, as shown in the right graph of Figure 11. Moreover, the increased friction coefficient in micro rolling, especially the ASR, lead to high plastic strain and the effective grain refinement [46], in which the high-angle grain boundaries were predominant, and was consistent consisted with the result in Figure 6b. 

**Figure 13:**
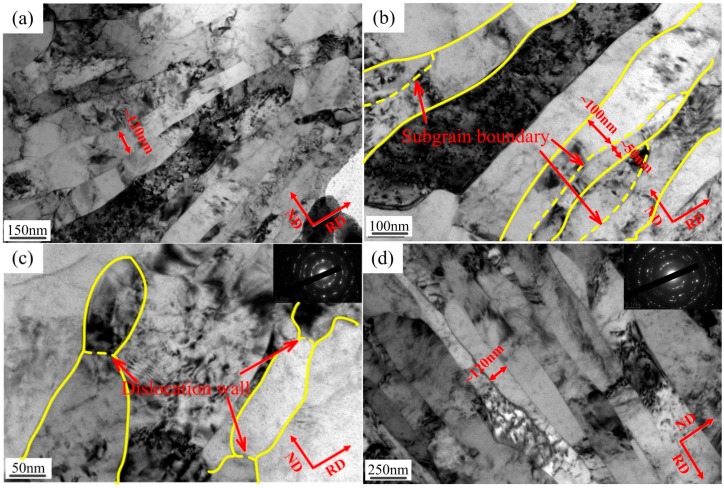
TEM bright-field micrograph of (**a**) thin lath structure formed; (**b**) lamella longitudinal splitting; (**c**) long laths breaking down into subgrains (thickness 76 μm); and (**d**) final grain refinement (thickness 25 μm) by ASR + micro rolling.

**Figure 14:**
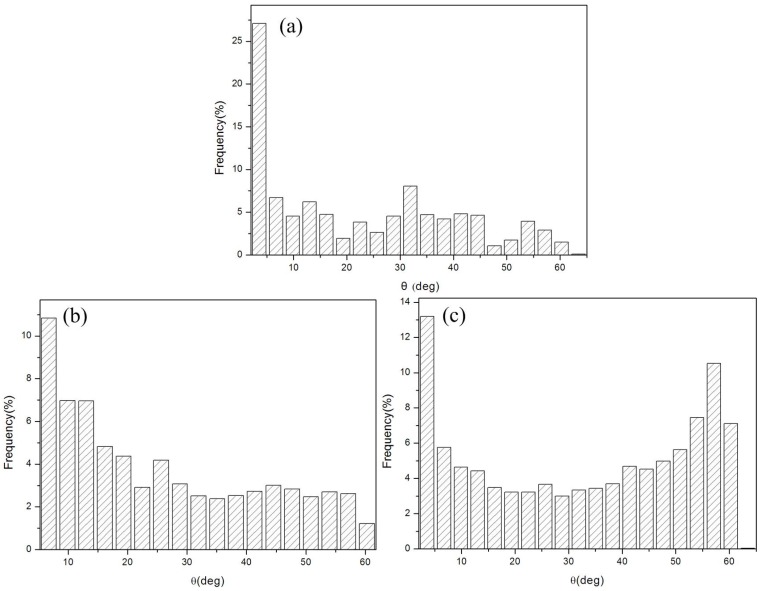
Distribution of angle between the (sub)grains of pure copper with thicknesses of (**a**) 5800 μm; (**b**) 76 μm; and (**c**) 25 μm.

With respect to the size effect of ultimate strength, some variance in microstructure for pure copper samples of different thicknesses was expected, in particular for the grain refinement (e.g., change of grain sizes) and the dislocation density. The TEM investigation shown in Figure 13 provided important information on the mechanism of microstructure evolution due to ASR + micro rolling. Ultra-high strain led to the formation and multiplication of ultrafine microstructures approximately parallel to the RD, which contain thin laths and elongated subgrain structures consisting of dislocation cells (Figure 13a). The grain sizes were refined from ~60 μm to ~120 nm subject to cold rolling to 76 μm. As a result of the improved shear deformation [47], the lath areas became curved, and some lath regions appear to have been locally extruded out to form a bulge (the lath boundaries and subgrain boundaries are marked by the yellow line and dotted line, respectively, in Figure 13b). It was suggested that the formation of a bulge tends to accelerate the lath splitting process in pure metal deformation [48]. Meanwhile, the dislocation accumulated at several locations to form a transverse dislocation wall as a “bamboo node” (some examples are marked by the red arrows in Figure 13c), which led to the breakdown of the laths into elongated segments [49]. The dimensions of those segments were ~80 nm wide and 200 nm long, which parallel to the RD. However, with further deformation, the grain refinement was non-significant, and the (sub)grain size was remind ~120 nm (Figure 13d, thickness 25 μm). With increased deformation, the dislocation walls received dislocations and increased their misorientations until they finally became transformed into high-angle boundaries (Figure 14), and it was shown that the fraction of high-angle boundaries increased when the sample thicknesses were decreased from 5800 μm to 25 μm. 

Considering the variation of ultimate strength, it can be roughly estimated using a standard model, such as the Hall–Petch relation for grain size (where ultimate strength is inversely proportional to square root of the grain size) and the Taylor model for the dislocation density (where ultimate strength proportional to the square root of the dislocation density) [50]. No pronounced size effect could be identified for the pure copper samples (with thicknesses from 5800 μm to 76 μm) subjected to cold rolling, that appeared to be (at least partially) responsible for the work hardening. However, with increased strain, that is, the thickness decreased from 76 μm to 25 μm, the pure copper appeared to be work softening. There were other effects which influenced ultimate strength and could be explained by the relaxed deformation constraint of grains at the surface—especially the very thin films—where there seemed to be additional hardening mechanisms dependent on the behavior of dislocation, such as the number of dislocation sources, active slip systems, and dislocation paths per thickness [16,51]. 

Therefore, to analyze the influence of size effect, the size dependent material constitutive model could be divided into two terms: the size dependent model and the size independent model. The model is expressed as [52,53]

(10) $$\left\{ \begin{array}{l} \sigma_{micro/macro}\left(\varepsilon \right)=\sigma_{ind}+\sigma_{dep}\\ \sigma_{ind}=M\tau_{R}\left(\varepsilon \right)+\frac{k\left(\varepsilon \right)}{\sqrt{d}}\\ \sigma_{dep}=\Gamma \left(m\tau_{R}\left(\varepsilon \right)-M\tau_{R}\left(\varepsilon \right)-\frac{k\left(\varepsilon \right)}{\sqrt{d}}\right) \end{array}\right.$$

where the $\sigma_{micro/macro}$ is the flow stress for material in micro/macro; ε represents the effective stain; $\sigma_{ind}$ and $\sigma_{dep}$ are size independent and size dependent of the flow stress, respectively. For FCC crystals, M was the orientation factor, equaled to 3.06 for the Taylor model. For a single crystal, m was the orientation factor (m ≥ 3). k(ε) is the locally intensified stress needed to propagate general yield across the polycrystal grain boundaries; d is the grain size.

The sample thickness, width and length were noted as h, b, and l, respectively. Therefore, the total volume of sample was

(11) $$V_{total}=h\times b\times l$$

**Figure 15:**
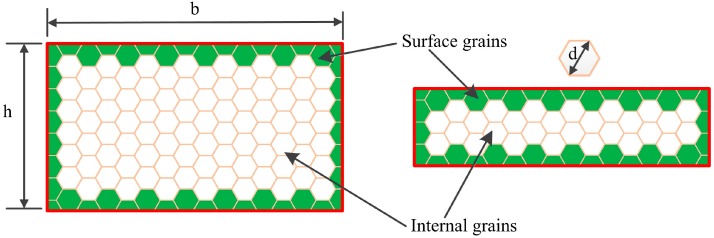
Surface grains and internal grains in sample.

The volume of surface grain in sample could be calculated

(12) $$V_{sur}=2d\times b\times l+2d\times h\times l+2d\times b\times h-8d^{3}$$

where Γ represented size factor, and the fraction of surface grains in samples, can be expressed as [43,53]

(13) $$\Gamma =\frac{2d\times b\times l+2d\times h\times l+2d\times b\times h-8d^{3}}{h\times b\times l}=2\frac{d}{h}+2\frac{d}{b}+2\frac{d}{l}-\frac{8d^{3}}{hbl}$$

where the h, b, and l are the thickness, width, and length, respectively. Figure 15 shows the surface and internal grains in the pure copper samples. Considering l,b≫d, the Equation (13) could be simplified

(14) $$\Gamma =2\frac{d}{h}$$

Furthermore, synthesizing each kind of situation, Γ was expressed as

(15) $$\Gamma =\left\{ \begin{array}{ll} 2, & d=h\\ 1, & d=\frac{h}{2}\\ 2\frac{d}{h}, & d<h\\ 0, & d\ll h \end{array}\right.$$

**Figure 16:**
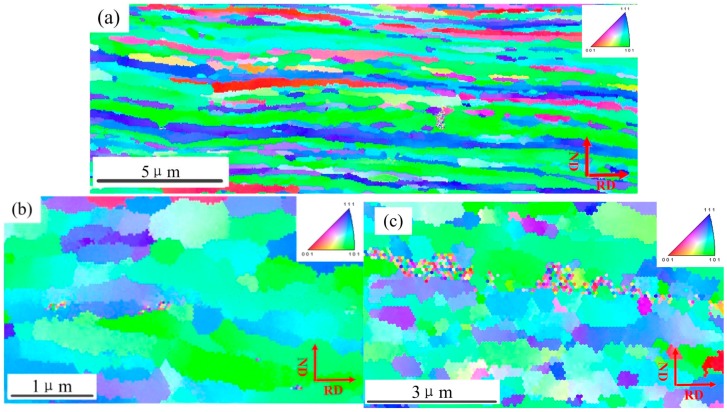
EBSD mapping representing the microstructure in the pure copper for thicknesses of (**a**) 76 μm and (**b**) 25 μm in internal and (**c**) 25 μm on surface.

It indicates that material behavior changes from single crystal to polycrystal material when the size factor varied from 2 to 0. As the thickness decreased, the ratio of surface grains increased significantly. In addition, in the TEM observation above, grain sizes were not further refined as thickness decreased from 76 μm to 25 μm. Furthermore, we found that the surface grains were refined to almost equiaxial crystals when internal grains size remained unchanged (Figure 16). This means that the surface grains increased significantly from 76 μm to 25 μm. 

Isolated dislocations frequently within the (sub)grains provided evidence that the plastic deformation within grain refinement occurred through the dislocation slip process. In the composite model, two regions were distinguished within the grains whereby the region at the grain boundaries was dominated by geometrically necessary dislocations (GNDs), whereas the core region in the interior of the grains was dominated by statistically stored dislocations (SSDs). The pure copper sample with the smaller grain size showed the higher ultimate strength, which related to the distance SSDs can move before they are trapped. Less strict dislocations for the surface grains led to a decrease of ultimate strength, and consisted of a dislocation density decrease from 76 μm to 25 μm.

It should be emphasized that a remarkable size effect on mechanical properties was investigated in the present work, that is, the ultimate strength of pure copper samples was increased from 5800 μm to 76 μm, but decreased from 76 μm to 25 μm after ASR + micro rolling at room temperature. That implied that as the increasing strain decreased and the thicknesses of the samples decreased, the material composite constitutive model considering surface factor should be applied as suggested by the authors. Thus, it seemed the size effect of pure copper by heavy cold rolling appear to bear a considerable resemblance to the micro manufacturing. Indeed, it has been suggested that the size effect in micro bending might actually be classified as an micro manufacturing process [54]. Thus, it seemed plausible that the size effect suggested in the present work may also applicable to the micro manufacturing process. 

## 5. Conclusions

The size effect on mechanical properties and texture in pure Cu foil during ASR rolling, was systematically investigated in this study. The following conclusions can be drawn.

The uniaxial tensile tests were conducted on the different thicknesses of 300, 200, 150, 100, 76, 50, and 25 μm. The ultimate strength of pure copper first increased from 5800 μm to 100 μm; however, it decreased from 76 μm to 25 μm. The reason was due to the microstructure, dislocation density, and rolling texture. It was found that grain sizes were refined from ~60 μm to ~120 nm with high angle boundaries. The dislocation density and micro strain were increased from 300 μm to 76 μm, and decreased from 76 μm to 25 μm. The rolling textures consisted of copper (C) {112}<111>, brass (Bs) {011}<211>, and S {123}<634>. 

The friction coefficient increased due to the sample thickness scale down in the micro rolling, that improved the asymmetrical rolling, which led to large plastic strain and the effective grain refinement. However, the grain size remained at ~120 nm from 76 μm to 25 μm. This increased the surface ratio, which was an important factor in the constitutive model which was considered size dependent. High surface ration meant more surface grain in the sample with less strict in dislocation slip, led to a decrease in ultimate strength. These findings arising from this research provide a basis understanding of deformation in micro manufacturing process.

## Figures and Tables

**Table 1:** Chemical composition of commercial pure copper (wt %).

Cu + Ag	Bi	Sb	As	Fe	Pb	S
≥99.90	0.001	0.002	0.002	0.005	0.005	0.005

**Table 2:** Surface roughness of commercial pure copper (wt %).

Sample (μm)	300	200	76	50	25
Surface Roughness (μm)	0.046 ± 0.006	0.052 ± 0.007	0.061 ± 0.007	0.065 ± 0.009	0.072 ± 0.01
